# Degeneration of Aortic Valves in a Bioreactor System with Pulsatile Flow

**DOI:** 10.3390/biomedicines9050462

**Published:** 2021-04-23

**Authors:** Naima Niazy, Mareike Barth, Jessica I. Selig, Sabine Feichtner, Babak Shakiba, Asya Candan, Alexander Albert, Karlheinz Preuß, Artur Lichtenberg, Payam Akhyari

**Affiliations:** 1 Department of Cardiac Surgery, Medical Faculty, University Hospital Düsseldorf, Heinrich-Heine-University Düsseldorf, Moorenstraße 5, 40225 Düsseldorf, Germany; niazy.naima@gmail.com (N.N.); mareike.barth@med.uni-duesseldorf.de (M.B.); jessica.selig@med.uni-duesseldorf.de (J.I.S.); sabine.feichtner@hotmail.de (S.F.); babak.shakiba@hhu.de (B.S.); asya.candan@hhu.de (A.C.); Alexander.Albert@klinikumdo.de (A.A.); payam.akhyari@med.uni-duesseldorf.de (P.A.); 2 Department of Cardiovascular Surgery, Klinikum Dortmund gGmbH, Beurhausstraße 40, 44137 Dortmund, Germany; 3 Faculty of Biotechnology, Bioprocessing, Modulation and Simulation, University of Applied Sciences Mannheim, Paul-Wittsack-Straße 10, 68163 Mannheim, Germany; k.preuss@hs-mannheim.de

**Keywords:** calcific aortic valve disease, degeneration, bioreactor system, tissue cultivation, ECM remodeling

## Abstract

Calcific aortic valve disease is the most common valvular heart disease in industrialized countries. Pulsatile pressure, sheer and bending stress promote initiation and progression of aortic valve degeneration. The aim of this work is to establish an ex vivo model to study the therein involved processes. Ovine aortic roots bearing aortic valve leaflets were cultivated in an elaborated bioreactor system with pulsatile flow, physiological temperature, and controlled pressure and pH values. Standard and pro-degenerative treatment were studied regarding the impact on morphology, calcification, and gene expression. In particular, differentiation, matrix remodeling, and degeneration were also compared to a static cultivation model. Bioreactor cultivation led to shrinking and thickening of the valve leaflets compared to native leaflets while gross morphology and the presence of valvular interstitial cells were preserved. Degenerative conditions induced considerable leaflet calcification. In comparison to static cultivation, collagen gene expression was stable under bioreactor cultivation, whereas expression of hypoxia-related markers was increased. Osteopontin gene expression was differentially altered compared to protein expression, indicating an enhanced protein turnover. The present ex vivo model is an adequate and effective system to analyze aortic valve degeneration under controlled physiological conditions without the need of additional growth factors.

## 1. Introduction

Calcific aortic valve disease (CAVD) is the most common valvular heart disease in developed countries. CAVD is characterized by thickening of the valve leaflets and subsequent tissue calcification resulting in functional aortic stenosis leading to heart failure and death if not treated. Other factors contributing to CAVD development are well defined cardiovascular risk factors, e.g., hypertension, smoking, obesity, and diabetes [1]. Presently, surgical or trans-catheter valve replacement is the only established treatment [2]. On the mechanistic level, CAVD is an active biological process characterized by leaflet stiffening, formation of extracellular calcified nodules [3], altered extracellular matrix (ECM) turnover [4], and phenotype changes in valvular interstitial cells (VIC) [5,6,7]. Under physiologic conditions in vivo, the aortic valve is exposed to a complex environment of cyclic tensile, shear and flexural stress inducing strain in circumferential and radial direction. Due to its anatomical position it is exposed to higher pressure and shear stress values than other heart valves [8]. VIC proliferation, collagen synthesis, and calcification are known to be affected by strain state and flexural deformation of the leaflet [9,10,11]. Ex vivo models showed that flow alone can promote differentiation of fibroblasts into activated myofibroblasts [10], and stretching of valves results in calcification and expression of pro-degenerative markers [12]. Aortic valve regurgitation likewise results in changes in gene expression common to those occurring in CAVD progression [13]. Moreover, abnormal fluid sheer stress frequencies induce the expression of pro-degenerative markers and enhance the expression of matrix metalloproteinase 2 and 9 (MMP2 and MMP9) and their activity [14]. However, under experimental settings biaxial stretching may result in preservation of native micro ECM structure [15]. Examinations of individual leaflets or parts thereof make up the majority of settings in previous studies. However, these settings do not fully represent the conditions of valve deformation during physiologic cyclic loading [8,12,15,16]. Therefore, alternative model systems are required that reflect physiological conditions during circular opening and closing due to pulsatile flow through the valve. Nevertheless, bioreactors allowing pulsatile flow through an intact aortic valve are mainly known in the context of tissue engineering and therefore used in experiments involving tissue engineered bioprosthetic devices or decellularized conduits [17,18]. Hence in the presented work, a computer-controlled bioreactor system is presented that allows cultivation of valve bearing native aortic roots under controlled physiological conditions and pulsatile flow through the valve as an adequate and effective environment to analyze aortic valve degeneration.

## 2. Materials and Methods

### 2.1. Bioreactor Design

**Figure 1:**
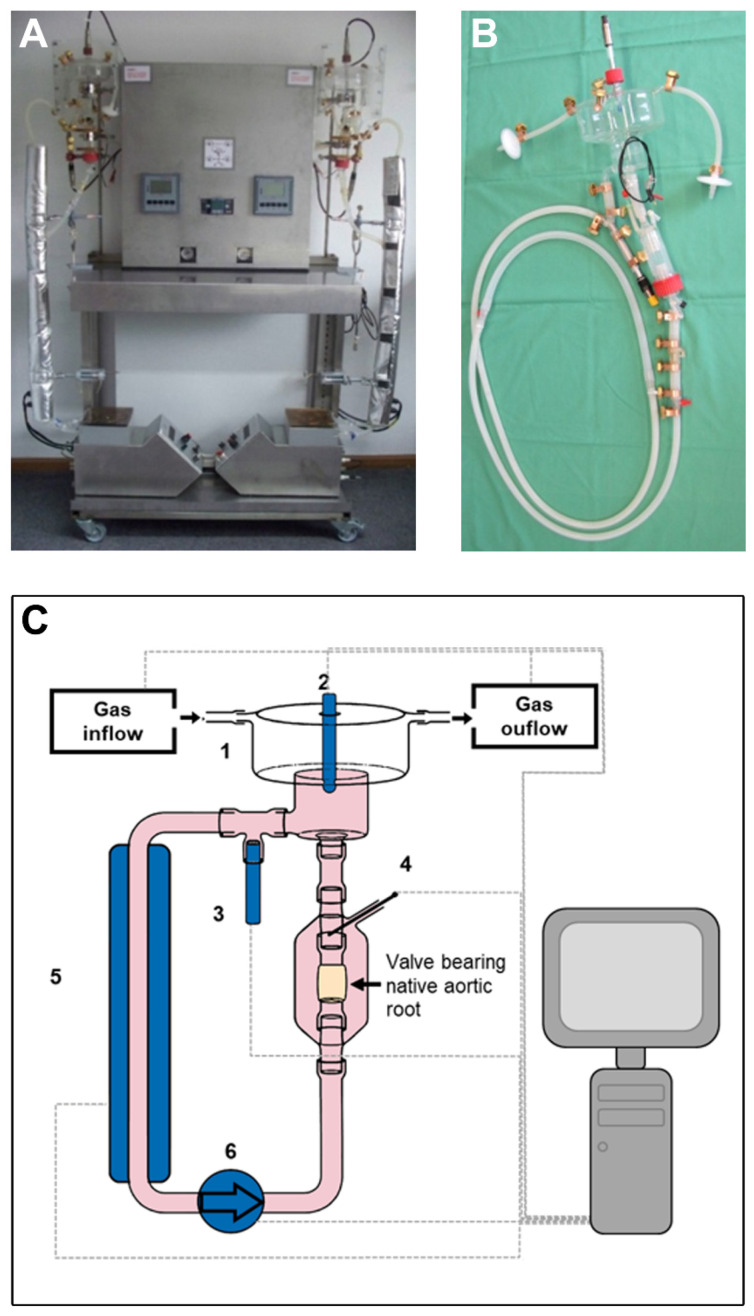
Computer-controlled bioreactor system with pulsatile flow. (**A**,**B**) The bioreactor system consists of computer-controlled regulatory elements and a tube system. (**C**) Schematic overview of the bioreactor system. Gas exchange occurs by surface aeration in the oxygenation camber (1). pH level (2), oxygen saturation (3), and pressure are measured and maintained via quantity and composition of gas inflow. Temperature (4) is measured and regulated by heating sleeve (5). Perfusion medium is piped through the heart valve by a pulsatile roller pump (6).

The bioreactor system consists of a tube system and external components for processing of sensor input and regulation of function (Figure 1A). The herein presented bioreactor is an elaborated version of a custom-made prototype bioreactor system that has been previously developed and used for tissue engineering purposes [19]. The bioreactor circuit is composed of a heart valve chamber that harbors the valve bearing native aortic root including the aortic valve. Moreover, the set-up consists of an oxygenation chamber as well as individual sensor probes for online measurement of pH level (EasyFerm Plus PHI VP120 Pt 100, Hamilton Company, Bonaduz, Switzerland; Stratos E 2402 pH, Knick, Berlin, Germany), O_2_ saturation (VisiFerm DO 120, Hamilton Company), atmospheric pressure in the oxygenation chamber (Type 8611 eControl, Bürkert Fluid Control Systems, Ingelfingen, Germany) and temperature (Pt100 Class B, custom designed by Engineo, Kelkheim, Germany) (Figure 1B, schematic overview in Figure 1C). According to user determined settings in the process control software values for temperature (37 °C), pH value (7.35) and chamber pressure (107 mbar corresponding to 80 mmHg) were maintained throughout the cultivation period. Oxygen levels in the circulating medium were recorded and saturation was around 100%. The bioreactor system is entirely controlled by an automated custom designed software system (Engineo). Circulating culture medium was perfused through the heart valve by a pulsatile roller pump (LivaNova, formerly Stöckert, London, UK) with 50 rounds per minute (generating a flow of 2.2 L/min) in the herein described experiments. Pressure was regulated through gas flow and pH level was adjusted by the flexible changing of CO_2_ supply, again in a fully automated, software-guided pattern. Gas exchange occurred by surface aeration in the oxygenation camber and temperature was regulated by a heating sleeve covering a large part of the perfusion circuit (Figure 1A).

### 2.2. Cultivation of Valve Bearing Native Aortic Roots

Valve bearing native aortic roots containing a small rim of adjacent myocardial tissue was dissected from fresh ovine hearts obtained from a local abattoir (Laame, Wuppertal, Germany). Next, valve bearing native aortic roots was washed in PBS with 200 U/mL penicillin (Thermo Fisher Scientific, Waltham, MA, USA), 200 µg/mL streptomycin (Thermo Fisher Scientific), and 5 µg/mL amphotericin B (Thermo Fisher Scientific) and sewn into the heart valve camber of the bioreactor system under sterile conditions within a conventional flow chamber. The bioreactor circuit (containing oxygen level, pH, and temperature probes) was filled with cell culture medium and connected to the bioreactor system. For standard cultivation (BR-std) DMEM with 4.5 mg/mL glucose and GlutaMAX (Thermo Fisher Scientific) was used containing 10% fetal calf serum (Sigma-Aldrich, Munich, Germany), non-essential amino acids (Sigma-Aldrich), 200 U/mL penicillin, 200 µg/mL streptomycin, and 5 µg/mL amphotericin B. For pro-degenerative medium conditions (BR-deg), standard cultivation medium was supplemented with 10 mM β-glycerophosphate (Sigma-Aldrich) and 1.5 mM calcium chloride (Carl Roth, Karlsruhe, Germany). After seven days of sterile bioreactor cultivation individual aortic valve leaflets were harvested from the aortic root and macroscopic images were taken using a commercial camera (Power Shot SX20IS, Canon, Tokyo, Japan) and a light pad (Slimlite LED 2447, Kaiser, Buchen, Germany). Aortic valve tissue samples were snap frozen in liquid nitrogen immediately at the end of the experiment and stored at −80 °C until further use.

For static cultivation valve bearing native aortic roots were dissected, washed, and then cultivated in 50 mL falcon tubes with loose cap in a cell culture incubator (37 °C, 5% CO_2_; Heracell CO_2_ incubator, Thermo Fisher Scientific). Cultivation was performed for seven days with a daily exchange of medium using above-mentioned standard and pro-degenerative conditions (CC-std and CC-deg).

### 2.3. Histological Staining and Immunohistochemistry

Frozen tissue sections of 5 μm thickness were fixed with 4% formalin and subjected to histological and immunohistological staining. Hematoxylin and eosin (H&E) staining was assessed for orientation and tissue integrity and Movat pentachrome staining was used to assess distribution of collagen, glycosaminoglycans, and elastic fibers within the valvular tissue. Biomineralization was confirmed via Alizarin red and von Kossa staining. Staining procedures were performed as previously described [20,21].

Immunohistological staining was performed as previously described [22] with antibodies targeting vimentin (Progen Biotechnik GmbH, Heidelberg, Germany) and von Willebrand factor (vWF; Agilent Technologies, Santa Clara, CA, USA). Subsequently, sections were stained with alexa488 and alexa594 conjugated secondary antibodies (Thermo Fisher Scientific). Sections were embedded with Vectashield with DAPI (Vector Laboratories, Burlingame, CA, USA). Images of all sections were taken using a Leica DM 2000 and LAS version 3.8 software (Leica Microsystems, Wetzlar, Germany).

### 2.4. RNA Isolation and Semi-Quantitative Real-Time PCR (qRT-PCR)

Aortic valve leaflets were homogenized and RNA was isolated using trizol chloroform extraction (TRI Reagent, Sigma-Aldrich) according to manufacturer’s instructions. Further purification of RNA and reverse transcription were performed using RNeasy Mini Kit (QIAGEN, Hilden, Germany) and QuantiTect Reverse Transcription Kit (QIAGEN) according to manufacturer’s specification. RNA integrity (RIN) was measured using Eukaryote Total RNA Nano Chip (Agilent Technologies), whereas all RIN-values were higher than 8.4. Sequence of intron-spanning primers for ribosomal protein L29 (*RPL29*; reference gene), osteopontin (*OPN*), collagen type 1 alpha 1 (*COL1A1*), alkaline phosphatase (*ALPL*), transforming growth factor beta (*TGFβ*), hypoxia-inducible factor 1 alpha (*HIF1α*), and vascular endothelial growth factor (*VEGF*) are listed in Table 1. Real-time quantitative amplification of RNA was performed using GoTaqR qPCR Master Mix (Promega, Madison, WI, USA) in a StepOnePlus Real Time PCR system according to the manufacturer’s instructions (Applied Biosystems Inc., Waltham, MA, USA). Relative gene expression levels were calculated using the delta-delta-CT method.

### 2.5. Western Blot Analysis

For protein isolation, frozen valve tissue samples were mechanically homogenized and sonicated for 10 min in RIPA buffer (Sigma-Aldrich) containing cOmplete protease inhibitor cocktail (Sigma-Aldrich). Samples were separated on 10% SDS-PAGE gels and transferred to nitrocellulose membrane (Bio-Rad Laboratories, Munich, Germany) via Western blotting. After blocking for 1 h with 5% bovine serum albumin (Sigma-Aldrich) in tris-buffered saline with Tween20 (TBST; 50 mM Tris-Cl [Sigma-Aldrich], 150 mM NaCl [Merck, Darmstadt, Germany], 0.1% Tween20 [Merck]), membranes were incubated with primary antibodies specific against alpha smooth muscle actin (αSMA; Sigma-Aldrich), OPN (Biozol, Eching, Germany) or alpha-tubulin (Sigma-Aldrich). Membranes were washed with TBST and incubated with horseradish peroxidase–conjugated secondary antibodies (Jackson ImmunoResearch, Cambridgeshire, UK; Dianova, Hamburg, Germany) and bands were visualized using WesternBright Chemilumineszenz Substrat Quantum (Advansta, Menlo Park, CA, USA) with an Amersham Imager 600 (GE Healthcare, Chicago, IL, USA).

### 2.6. Statistical Analysis

Data are presented as mean and standard error of mean. Significance was determined with nonparametric testing. Mann-Whitney U test was performed for pairwise comparison of standard and pro-degenerative medium conditions, while Kruskal-Wallis-Test with Dunn’s multiple comparisons post-hoc test was used to compare native, BR, and CC culture conditions, respectively. All datasets were analyzed using GraphPad Prism version 5.01 (GraphPad Software, La Jolla, CA, USA). *p*-values < 0.05 were considered as statistically significant.

## 3. Results

### 3.1. Bioreactor Function and Macroscopic Analysis

**Figure 2:**
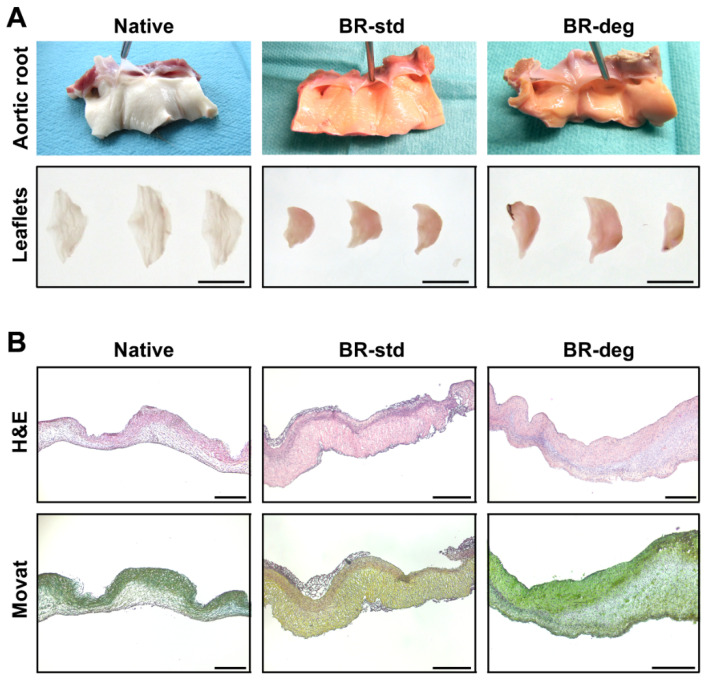
Macroscopic and histological analysis of ovine aortic valve conduits. Valve bearing native aortic roots were cultivated in a bioreactor system with pulsatile flow for one week. (**A**) Macroscopic analysis after bioreactor cultivation shows no remarkable differences between aortic roots cultivated in standard (BR-std) or pro-degenerative medium containing β-glycerophosphate and calcium chloride (BR-deg), but a thickening of leaflets compared to native valve bearing aortic roots (Native). Bars: 1 cm. (**B**) Overall leaflet structure (H&E staining, upper panel) and three-layer structure of extracellular matrix (Movat pentachrome staining, lower panel) remained after bioreactor cultivation. Representative images of eight replicates are presented. Bars: 400 µm.

The bioreactor system demonstrated continuous and constant operation with maintained physiological parameters throughout each experimental run. At the time of explantation out of the bioreactor chamber, the aortic valve leaflets appeared smaller and thicker for both, BR-std and BR-deg, as compared to native leaflets (Figure 2A, upper panel). Likewise, subsequent lumination through the leaflets showed that leaflets were less translucent after bioreactor cultivation compared to native aortic valve leaflets (Figure 2A, lower panel). Leaflets presented intact structure upon macroscopic examination, with the entire aortic root tissue being free of visible sclerosis after one week of bioreactor-stimulated culture. However, during the experimental cultivation medium color led to slight reddish discoloration after cultivation compared with native control tissue.

On histological level, leaflets displayed intact morphology as determined by H&E staining (Figure 2B, upper panel). Cell density in the spongiosa varied within biological replicates in all groups ranging from densely populated to a sponge-like appearance with few cells. Other than that, H&E staining showed a homogeneous cellularity within the leaflet layers. Based on Movat pentachrome staining a three-layered structure of the aortic leaflet tissue was preserved (Figure 2B, lower panel). The aforementioned leaflet thickening without major disruption of microarchitecture was observed in all analyzed specimens regardless of the respective medium condition.

### 3.2. Analysis of Tissue Biomineralization and Cell Composition

**Figure 3:**
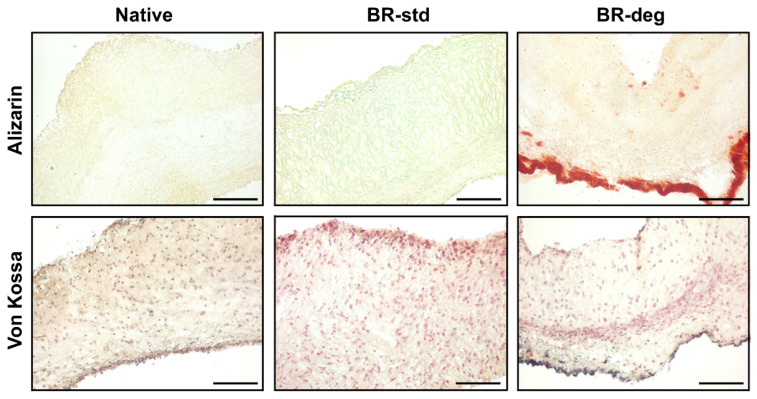
Ex vivo cultivation in pro-degenerative conditions led to biomineralization. Cross-sections of native aortic valve leaflets (Native) and leaflets cultivated under standard (BR-std) or pro-degenerative conditions (BR-deg) were stained with Alizarin red (upper panel) and von Kossa (lower panel). Alizarin red staining (red) and von Kossa staining (black) indicating biomineralization, were detected after cultivation in BR-deg, but not in BR-std or native control. Representative pictures of eight replicates were chosen. Bars: 100 µm.

In order to assess areas of biomineralization two different histological stainings were applied, Alizarin red and von Kossa staining (Figure 3). In native control and BR-std no biomineralization was detected (Figure 3, left and middle columns). In contrast, under pro-degenerative conditions considerable biomineralization arose in cells of the lamina ventricularis (Figure 3, right column). Small amounts of biomineralization could also be detected in the lamina fibrosa.

**Figure 4:**
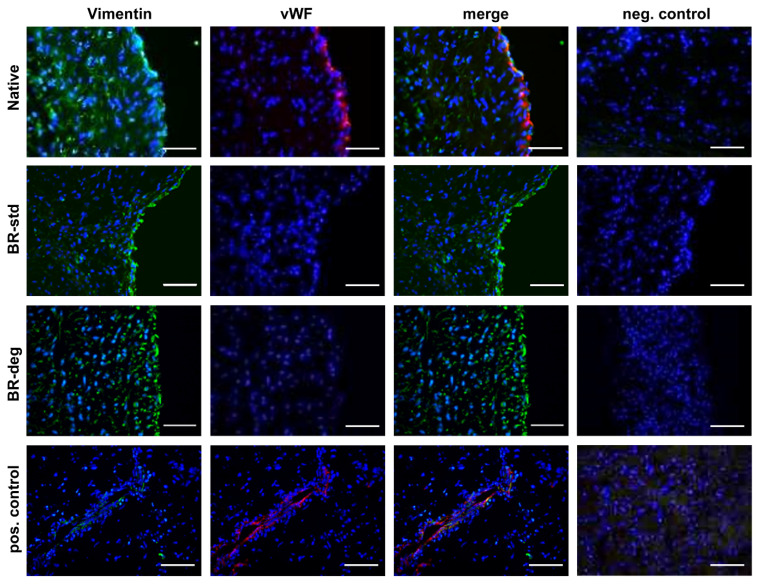
Cellular composition of the aortic valve after bioreactor cultivation. Immune staining with vimentin (green) and vWF (red) specific antibodies was performed on aortic valve cross-sections. Vimentin positive cells were detected in native tissue (first panel) as well as after one week of bioreactor cultivation in standard (BR-std, second panel) pro-degenerative medium (BR-deg, third panel). vWF staining is visible in native aortic valve leaflets (Native, first panel), but not after bioreactor cultivation (second and third panel). Nuclei were counterstained with DAPI. Blood vessels in myocardial specimens served as positive staining control (last panel). Negative control was performed by staining without primary antibodies (right column). Representative pictures of five replicates were chosen. Bars: 50 µm.

In order to characterize the morphology of cells populating the aortic valve tissue after bioreactor cultivation immunohistological staining was applied (Figure 4). Vimentin staining was detected in all layers of native aortic valve leaflets and remained detectable after bioreactor cultivation (Figure 4, left column). In addition, vWF-specific staining was traceable in the endothelial cell layer of native samples lining the aortic valve leaflet on the ventricular and the aortic side (Figure 4, middle column). However, after bioreactor cultivation vWF-specific staining is lost.

### 3.3. Gene Expression of Pro-Degenerative Markers

**Figure 5:**
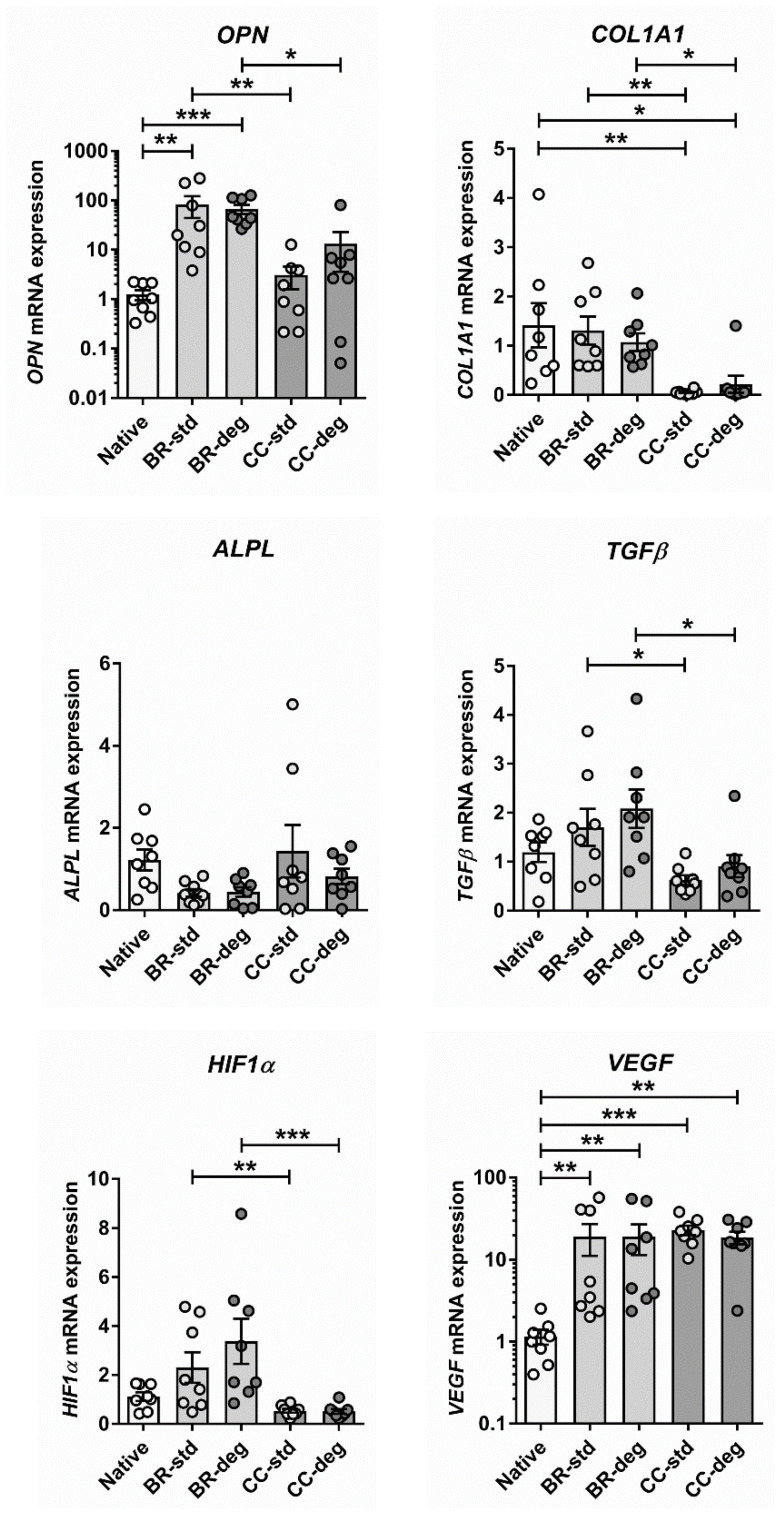
Characterization of the gene expression in aortic valve after bioreactor cultivation compared to static conditions. Valve bearing native aortic roots were cultivated in bioreactor with pulsatile flow (BR) or in a cell culture incubator (CC) for one week using standard medium (BR-std and CC-std) or medium with pro-degenerative conditions (BR-deg and CC-deg). Ovine aortic valve samples were examined using qRT-PCR with specific primers against the housekeeper RLP29, the pro-degenerative markers osteopontin (*OPN*), collagen type I (*COL1A1*), alkaline phosphatase (*ALPL*), and *TGFβ* as well as *HIF1α* and *VEGF*. Values are shown as means and standard error of means, dots indicate eight biological replicates. For statistical analysis Mann–Whitney test was performed to compare standard and pro-degenerative conditions and Kruskal–Wallis test was used to compare native, BR and CC cultivation. Stars indicate significant differences in Dunn’s multiple comparison post-hoc test (* *p* < 0.05; ** *p* < 0.01; *** *p* < 0.001).

To characterize the effects of bioreactor cultivation with pulsatile medium flow and different medium conditions, gene expression analysis targeting established markers of aortic valve degeneration was performed. The effect of bioreactor cultivation with pulsatile flow was compared to static cultivation and to native aortic valve tissue (Figure 5). Bioreactor cultivation led to a significant increase of *OPN* expression compared to native aortic valve control (native vs. BR-std: *p* = 0.0030; native vs. BR-deg: *p* = 0.0005) and static cultivation (BR-std vs. CC-std: *p* = 0.0090; BR-deg vs. CC-deg: *p* = 0.0401). However, expression after static cultivation was not significantly altered compared to native control (native vs. CC-std: *p* ˃ 0.9999, native vs. CC-deg: *p* = 0.5727).

In contrast, *COL1A1* expression was not altered by bioreactor cultivation compared to native control (native vs. BR-std: *p* ˃ 0.9999; native vs. BR-deg: *p* ˃ 0.9999), but significantly increased compared to static cultivation of the leaflet tissue (BR-std vs. CC-std: *p* = 0.0014, BR-deg vs. CC-deg: *p* = 0.0157). *COL1A1* expression was also significantly increased under static cultivation compared to native control (native vs. CC-std: *p* = 0.0030; native vs. CC-deg: *p* = 0.0141). Gene expression of *ALPL* was not significantly altered between culture conditions neither under standard conditions (native vs. BR-std: *p* = 0.1018; native vs. CC-std: *p* ˃ 0.9999; BR-std vs. CC-std: *p* = 0.6095) nor under pro-degenerative conditions (native vs. BR-deg: *p* = 0.0779; native vs. CC-deg: *p* = 0.7301; BR-deg vs. CC-deg: *p* = 0.8667). However, in degenerative condition a trend toward the downregulation of *ALPL* in BR-deg compared to native control was observed (*p* = 0.0779).

Moreover, gene expression levels of three key players in neovascularization, namely *TGFβ*, *HIF1α,* and *VEGF*, the latter being regulated by *HIF1α*, were analyzed (Figure 5, lower panel). *TGFβ* showed no significant changes neither in bioreactor cultivation (native vs. BR-std: *p* ˃ 0.9999; native vs. BR-deg: *p* = 0.2899) nor in static cultivation compared to native control (native vs. CC-std: *p* = 0.1211; native vs. CC-deg: *p* ˃ 0.9999). Nevertheless, *TGFβ* expression was increased under static cultivation compared to bioreactor cultivation (BR-std vs. CC-std: *p* = 0.0267; BR-deg vs. CC-deg: *p* = 0.0296). Likewise, *HIF1α* expression was significantly increased after bioreactor cultivation compared to static cultivation (BR-std vs. CC-std: *p* = 0.0063; BR-deg vs. CC-deg: *p* = 0.0005). Furthermore, *HIF1α* was by trend downregulated in CC-std when compared to native valve tissue (native vs. CC-std: *p* = 0.0779). Interestingly, *VEGF* expression was significantly increased under bioreactor cultivation (native vs. BR-std: *p* = 0.0090; native vs. BR-deg: *p* = 0.0071) as well as under static cultivation (native vs. CC-std: *p* = 0.0009; native vs. CC-deg: *p* = 0.0012) compared to native controls, respectively. A direct comparison of both cultivation systems did not reveal differences in *VEGF* expression (BR-std vs. CC-std: *p* ˃ 0.9999; BR-deg. vs. CC-deg: *p* ˃ 0.9999).

### 3.4. Bioreactor Mediated Changes on the Protein Level

**Figure 6:**
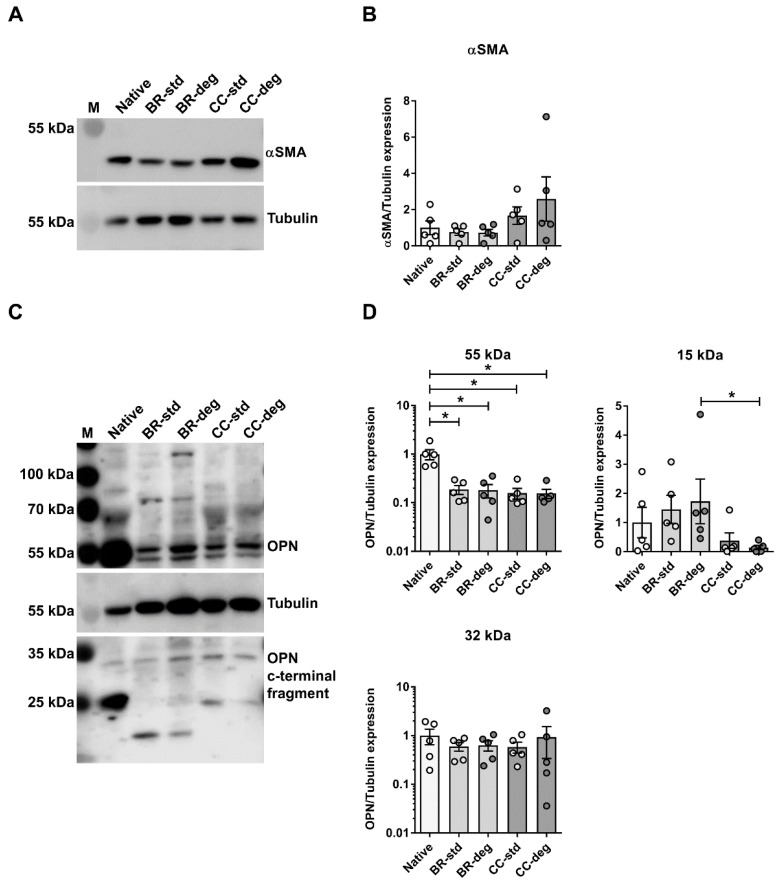
Bioreactor cultivation led to altered OPN protein levels. Lysates were analyzed by Western blot with specific antibodies for (**A**,**B**) αSMA, (**C**,**D**) osteopontin (OPN), and Tubulin as loading control. Positions of molecular weight markers are indicated on the **left**. (**B**,**D**) Graphic representation of band intensities is displayed on the right, using means and standard error of means (*n* = 5). For OPN protein bands at approximately 55 kDa and 32 kDa were subjected to intensity analysis. Kruskal–Wallis test was performed and significance of Dunn’s post-test is indicated with stars (* *p* < 0.05). Control: native leaflets, BR: bioreactor cultivation, CC: static cultivation in cell culture, std: using standard medium, deg: using pro-degenerative medium.

To assess the protein level Western blot analysis was performed (Figure 6; for original images of Western blot membranes see Supporting Information). Protein level of αSMA, a marker of VIC differentiation into activated myofibroblast phenotype, was not significantly altered after bioreactor cultivation or static cultivation (Figure 6A,B). Western blot analysis of OPN expression displayed several bands. Expression of 55 kDa OPN was significantly higher in native aortic valves compared to bioreactor cultivation (native vs. BR-std: *p* = 0.0486; native vs. BR-deg: *p* = 0.0327) and static cultivation (native vs. CC-std: *p* = 0.0112; native vs. CC-deg: *p* = 0.0175). In contrast, expression of 32 kDa OPN C-terminal fragment was unaltered. Expression of 15 kDa C-terminal OPN was significantly higher in bioreactor cultivation compared to static cultivation under pro-degenerative conditions (BR-deg vs. CC-deg: *p* = 0.0267) (Figure 6C,D).

## 4. Discussion

In the present study, we address aortic valve degeneration with a novel ex vivo model. Our data deliver the following insights: (1) The introduced bioreactor system facilitates the induction of biomineralization in aortic valves, without the need of stimulation with pro-degenerative growth factors. (2) Cultivation in pulsatile flow partly prevents altered gene expression due to ex vivo cultivation. (3) OPN expression level and post-translational modification patterns are modified by controlled pulsatile flow.

### 4.1. Induction of Biomineralization Due to Bioreactor Cultivation

The herein presented bioreactor is an effective system to induce biomineralization without a profound manipulation like external TGFβ treatment. TGFβ has been previously used to induce calcification in former bioreactor models [10,23] and triggers degenerative processes leading to enhanced calcification in CAVD [24,25]. However, TGFβ also has impact on VIC differentiation, ECM production, and nodule formation [26,27,28,29]. Cultivation in the introduced bioreactor system induces biomineralization using pro-degenerative medium without the need of growth factor addition. This process might be triggered by the general loss of the endothelial cell layer during bioreactor cultivation due to the lack of specific endothelial supporting supplements, shown by staining with endothelial cell marker vWF. Loss of endothelial cells as a result of endothelial dysfunction, inflammation, or endothelial-mesenchymal-transition is a hallmark of early aortic valve degeneration [12,30]. Our observation of significantly higher *OPN* expression under bioreactor cultivation validate this and might favor biomineralization under pro-degenerative medium. Ceasing integrity of the endothelial cell layer triggers VIC activation remodeling and calcium deposition [1]. In bioreactor cultivation, endothelial cell growth may be reduced, since cultivation medium did not contain growth factors commonly used to maintain endothelial cell proliferation. On the other hand, supplements containing growth factors could impact VIC behavior and therefore biomineralization, as mentioned above.

Furthermore, the endothelial cell layer may be disrupted during cultivation after rapid increase of the flow rate in the employed bioreactor system [20]. In our model loss of endothelial cells and biomineralization is visible in both fibrosa and ventricularis, whereas in CAVD valve calcification more frequently occurs on the aortic side of leaflet as opposed to the fibrosa [31,32,33]. In the fibrosa, the regional wall sheer stress distribution shows more heterogeneity and is more dependent on the valve morphology and the specific phase of the cardiac cycle as compared to the ventricularis layer, making the layer more vulnerable to degeneration [34,35]. Even though pulsatile flow during bioreactor cultivation partly mimics native flow patterns, it may not perfectly adapt distribution of wall sheer stress levels that are present in vivo. However, ECM structure remains intact and homogeneous cellularity within the leaflet layers is preserved, indicating sufficient nutrient transport during bioreactor cultivation.

### 4.2. Bioreactor Cultivation Leads to Altered Gene Expression Patterns

Ex vivo cultivation of dissected aortic valve tissue leads to leaflet thickening and significant changes in gene expression, both, in the bioreactor system and during static cultivation in the cell culture incubator. In our bioreactor model, leaflets remain in the native aortic root during cultivation, which allows biomimetic cultivation using pulsatile flow.

However, bioreactor cultivation leads to visible thickening of aortic valve leaflets and increased expression levels of *VEGF*, which plays a key role in neoangiogenesis. While healthy aortic leaflets are translucent and oxygen supply occurs via diffusion, fibrotic thickening leads to neovascularization during clinically occurring CAVD. This may be due to local hypoxia [36]. Both *VEGF* and *HIF1α* are significantly upregulated in degenerating human aortic valves and expression occurs predominantly in the sites of calcification [37]. Regulation of *HIF1α* expression mainly occurs on protein level, where it is rapidly degenerated in normoxic conditions [38,39]. Interestingly, *HIF1α* gene expression after bioreactor cultivation is significantly higher than after static cultivation, indicating pulsatile flow may affect *HIF1α* expression on RNA level. However, the overall impact may be small since expression compared to native control is not significantly altered in both cultivation conditions.

A similar effect is observed for *TGFβ* expression: expression after bioreactor is significantly higher than after static cultivation, but both show no alteration compared to the native control. TGFβ is known to play a role in neoangiogenesis, but also affects VIC differentiation and ECM turnover [26,27,28,29].

Increasing expression of *TGFβ* enhances myofibroblast-like activation state of VIC in vitro [40]. Furthermore, mechanical stimuli alone may contribute to transition of VIC from a quiescent phenotype to a myofibroblast phenotype [41,42,43]. The latter activated myofibroblast-like state of VIC is characterized by elevated αSMA expression [40]. While isolated pathological stretch is known to increase αSMA expression [44], pressure on aortic valve leaflets has been demonstrated to decrease αSMA expression [45]. A previous study using an ex vivo stretch and pressure bioreactor has observed downregulation of αSMA protein expression [46]. Our results show no significant changes in αSMA expression after both bioreactor and static cultivation. However, scattering within the biological replicates is wider after static cultivation. Therefore, regulation of αSMA expression may be mechanosensitive, generating a more homogenous expression pattern even though overall expression is not altered. Since expression of αSMA is unaltered after bioreactor cultivation, an absent impact of bioreactor cultivation on *TGFβ* expression matches our other results.

In addition, gene expression levels of *ALPL* are unaltered after both static and bioreactor cultivation. *ALPL* is known to promote tissue mineralization via hydrolysis of diphosphate. Nevertheless, *ALPL* expression was shown to decrease during cultivation of aortic VIC in 2D and 3D [21,47]. Similar effects, though not statistically significant could be observed in our bioreactor model.

On the other hand, *COL1A1* expression is preserved in bioreactor cultivation compared to native control but significantly reduced in static cultivation. Collagen is an important component of the ECM that provides structural and functional characteristics to valve tissue including mechanical integrity and biological signaling. Tension, circular, and biaxial stress stimulate collagen expression and increase collagen fiber alignment along the principal directions of strain [11,44,48]. Increased collagen expression however remains insignificant depending on the chosen read-out analysis [15]. Therefore, mechanical stimulation is likely to be the cause of preservation of *COL1A1* gene expression levels during bioreactor cultivation.

Taken together these results indicate that ex vivo cultivation per se leads to significant changes in gene expression though changes alter between static and bioreactor cultivation.

### 4.3. Altered Expression of the Osteogenic Marker OPN

In our study, we measured *OPN* gene expression and protein levels of full-length protein as well as C-terminal OPN fragments. Full length OPN is extensively phosphorylated and glycosylated leading to a shift in molecular weight (55–70 kDa) [49]. Phosphorylation of OPN is required for its protective impact on vascular calcification [50,51]. Moreover OPN polymeric complexes formed through crosslinking by transglutaminase 2 are altered in functional properties compared to OPN monomers [52,53]. Intensity of 55 kDa OPN bands shows significantly higher expression in native aortic leaflets compared to bioreactor or static cultivation, indicating higher expression of full length OPN in native leaflets. On the other hand OPN is cleaved by several matrix metalloproteases and thrombin into a N-terminal and an integrin binding C-terminal fragment [54,55]. In our setting the amount of detected C-terminal OPN fragment (approx. 32 kDa) is not significantly altered between native, bioreactor, and static condition. However, the amount of 15 kDa fragment, a further cleaved fragment of C-terminal OPN, is increased after bioreactor cultivation and significantly higher in BR-deg compared to CC-deg. In standard condition, differences remain insignificant due to high standard deviation within the groups. *OPN* gene expression is highly elevated in BR conditions compared to native valve, while protein levels are reduced for full-length protein and constant or increased, respectively, for degradation products. Overall, this result might hint at an altered turnover of OPN due to bioreactor cultivation.

A recent study comparing calcified and non-calcified human aortic valves observed elevated *OPN* RNA levels and elevated OPN plasma levels while OPN protein expression within the leaflet was significantly reduced in calcified valves of the examined patient group [56].

It is known that OPN intensively stains calcified regions in sclerotic aortic valves [57,58] but the functional role of OPN in CAVD is still controversial. OPN may bind to apatite crystals and is considered as a potential mineralization inhibitor [59] though at the same time OPN expression increases with the progression and calcification in human aortic valve tissue [60] suggesting a pro-degenerative role. Splice variants and post-translational modification may affect the role of OPN within disease progression [51,58]. Therefore, further analysis should aim to identify modality of OPN posttranslational modification and verify altered cleavage patterns due to cultivation in pulsatile flow.

## 5. Conclusions

In this study, we present a novel ex vivo model to analyze aortic valve degeneration under controlled physiological conditions and pulsatile flow while retaining ECM structure. Bioreactor cultivation under pro-degenerative conditions leads to considerable biomineralization within the valve tissue without requiring additional stimulation by cytokines or growth factors. Moreover, cultivation in pulsatile flow in part preserves *COL1A1* expression and modulates the expression of genes involved in response of hypoxia and also tissue protein expression of OPN. In conclusion, the established ex vivo model is an effective system to analyze aortic valve degeneration under controlled physiological conditions while retaining the morphological integrity.

## Figures and Tables

**Table 1:** Primer sequences used for qRT-PCR analysis.

Gene of Interest	Forward Primer	Reverse Primer
Ribosomal protein L29 (*RPL29*)	CCAAGTCCAAGAACCACACC	TATCGTTGTGATCGGGGTTT
Osteopontin (*OPN*)	GATGGCCGAGGTGATAGTGT	TCGTCTTCTTAGGTGCGTCA
Collagen type 1 alpha 1 (*COL1A1*)	AAGACATCCCACCAGTCACC	TAAGTTCGTCGCAGATCACG
Alkaline phosphatase (*ALPL*)	GACATCGCCTACCAGCTCAT	CACATCGGTTCTGTTCTTGG
Hypoxia-inducible factor 1 alpha (*HIF1α*)	GAAGCAAAGAATCCATTTTCCA	TGGTGACAACTGATCGAAGG
Transforming growth factor beta (*TGFβ*)	GAGCCAGAGGCGGACTACTA	TCGGACGTGTTGAAGAACAT
Vascular endothelial growth factor (*VEGF*)	CGGATCAAACCTCACCAAAG	AAATGCTTTCTCCGCTCTGA

## Data Availability

The data presented in this study are available on reasonable request from the corresponding author.
